# In-Flight Alignment Using *H*_*∞*_ Filter for Strapdown INS on Aircraft

**DOI:** 10.1155/2014/820305

**Published:** 2014-01-06

**Authors:** Fu-Jun Pei, Xuan Liu, Li Zhu

**Affiliations:** Beijing University of Technology, Beijing 100124, China

## Abstract

In-flight alignment is an effective way to improve the accuracy and speed of initial alignment for strapdown inertial navigation system (INS). During the aircraft flight, strapdown INS alignment was disturbed by lineal and angular movements of the aircraft. To deal with the disturbances in dynamic initial alignment, a novel alignment method for SINS is investigated in this paper. In this method, an initial alignment error model of SINS in the inertial frame is established. The observability of the system is discussed by piece-wise constant system (PWCS) theory and observable degree is computed by the singular value decomposition (SVD) theory. It is demonstrated that the system is completely observable, and all the system state parameters can be estimated by optimal filter. Then a *H*_*∞*_ filter was designed to resolve the uncertainty of measurement noise. The simulation results demonstrate that the proposed algorithm can reach a better accuracy under the dynamic disturbance condition.

## 1. Introduction

The strapdown inertial navigation system (SINS) necessitates an alignment stage to determine the initial condition before the navigation operation [1]. Since the initial alignment process directly relates to the accuracy and rapidness of SINS, it is vitally important. In recent years, a lot of researches have been developed for SINS alignment. Among which many kinds of filters have been designed to estimate the state of the SINS, such as Kalman filter (KF), extended Kalman filter (EKF), unscented Kalman filter (UKF), and *H*_*∞*_ filter.

Recent decades have witnessed the prosperity and maturity of aircraft navigation, and the SINS alignment problem under the dynamic condition has received detailed attention. Due to engine vibration, wind gusts, loading, fuel injection, and uncertain human impact, the aircraft is not absolutely stationary, so the output of the inertial sensors has errors with such external disturbances [2]. Therefore, the method of ground alignment cannot be used to perform the aircraft SINS alignment.

Conventional alignment methods including coarse alignment and fine alignment are widely used for SINS alignment. In the coarse alignment, the system attitude can be determined directly by using the known gravity and Earth rate signals in the local level frame and the measurements obtained by using accelerometers and gyros [3]. Then the Kalman filter is used for fine alignment in the conventional alignment method. However, under the dynamic disturbance condition, traditional alignment techniques cannot be used, because the gyros' measurements will include high rotational values (several orders of magnitude greater than the Earth's rotation rate) resulting from the aircraft's pitch and roll [4]. Therefore, in recent years a coarse alignment for SINS under dynamic interference condition has been investigated in [5, 6]. Referring to the coarse alignment method, the inertial frame alignment is adopted in [7] in the fine alignment process, and an adaptive Kalman filter is used. It can effectively depress the random disturbances in the velocity measurements caused by the ship rocking.

No matter what kind of filter is used, observability analysis of a dynamic system is needed before designing it to determine the efficiency of the filter. The observability of a linearized time-invariant system has extensively been studied in many early research works [8, 9]. The observability analysis of a linearized time-invariant system is straightforward by testing the rank of the observability matrix. While the observability analysis of a constant dynamic system is rather simple, the analysis of a time-varying system is quite cumbersome. A PWCS (piecewise constant system) theory was proposed in [10, 11] to simplify the analysis. In this method, the linear time-varying system was approximated by a piecewise constant model, and in each constant segment, a simplified null space test was performed to determine the observability properties [12]. At present, there are several ways for observability analysis of SINS. A state equation decoupling method is proposed in [13]. This method decomposes each state of the system equation into corresponding subspace to determine the observable state of the system. But it is only a qualitative analysis and it is strong subjective. Bar-Itzhack and Berman [14] evaluate the observability through eigenvalues of the covariance matrix. But this method cannot estimate the observability directly through state equation and measurement equation. It is performed after filtering, so there exists large amount of calculation and some other disadvantage.

In this paper, a novel alignment method is investigated and observability is analyzed by PWCS theory and SVD theory. Firstly, using the gravity in the inertial frame as a reference, the initial fine alignment error model of SINS is established. Then, on the one hand, the observability of SINS with 12 system state parameters is analyzed by PWCS theory. On the other hand, the observable degree of the system is computed by SVD theory. It can be seen that the system is completely observable; all the 12 system state parameters can be estimated. Finally, on the basis of observability, the filter can be designed to estimate the system state parameters. Due to the uncertainty of measurement noise characteristics, a fine alignment method based on *H*_*∞*_ filter is introduced. Simulation results show that this self-alignment scheme can effectively solve the alignment problem of SINS in dynamic interference environment.

This paper is organized as follows. A brief introduction to SINS error model in the inertial frame is presented in Section 2. The observability analysis of the SINS alignment based on the observability analysis of PWCS theory and SVD theory is analyzed in Section 3. In order to prove the analysis results, simulations are carried out in the Section 4. Conclusions are drawn in Section 5.

## 2. SINS Alignment Error Model in the Inertial Frame

Under the dynamic disturbance environment, the extra disturbed accelerations would invalidate the traditional alignment method. Therefore, a new alignment method for marine SINS using the gravity in the inertial frame as a reference has been discussed [15]. A fundamental aspect of inertial navigation is the precise definition of a number of Cartesian coordinate reference frames [16]. In this method, a special inertial frame, the initial time SINS body inertial frame (*i*_*b*0_ frame), is defined and is selected as transition reference frames in SINS alignment. The coarse alignment results are used as input for fine alignment; this section only gives a brief introduction to SINS error model in inertial frame.

In the fine alignment process, the initial attitude matrix *C*_*n*_^*b*^(*t*) can be represented as

(1) $$\begin{array}{c} C_{n}^{b}\left(t\right)=C_{e}^{n}C_{i}^{e}\left(t\right)C_{b}^{i}\left(t\right). \end{array}$$

According to the calculation method of coarse alignment scheme,

(2) $$\begin{array}{c} C_{e}^{n}=\left[\begin{array}{ccc} 0 & 1 & 0\\ -\sin L & 0 & \cos L\\ \cos L & 0 & \sin L \end{array}\right],\\ C_{i}^{e}\left(t\right)=\left[\begin{array}{ccc} \cos\omega_{ie}\left(t-t_{0}\right) & \sin\omega_{ie}\left(t-t_{0}\right) & 0\\ -\sin\omega_{ie}\left(t-t_{0}\right) & \cos\omega_{ie}\left(t-t_{0}\right) & 0\\ 0 & 0 & 1 \end{array}\right]. \end{array}$$

So, the key lies in the calculation of *C*_*b*_^*i*^(*t*):

(3) $$\begin{array}{c} C_{b}^{i}\left(t\right)={\left[C_{i}^{i^{\prime }}\left(t\right)\right]}^{-1}C_{b}^{i^{\prime }}\left(t\right), \end{array}$$

where

(4) $$\begin{array}{c} C_{b}^{i^{\prime }}\left(t\right)=C_{b}^{i^{\prime }}\left(0\right)\Delta C_{b}^{i}. \end{array}$$

The attitude matrix *C*_*b*_^*i*′^(0), which relates the body frame to the computed inertial frame at the beginning of the fine alignment, is derived from the coarse alignment. And Δ*C*_*b*_^*i*^ can be computed through the attitude quaternion algorithm.

Suppose the misalignment angles between inertial frame *i* and the computed frame *i*′ are

(5) $$\begin{array}{c} \varphi^{i}\left(t\right)={\left[\begin{array}{ccc} \varphi_{x}^{i}\left(t\right) & \varphi_{y}^{i}\left(t\right) & \varphi_{z}^{i}\left(t\right) \end{array}\right]}^{T}. \end{array}$$

So, *C*_*i*_^*i*′^(*t*) in (3) can be described as

(6) $$\begin{array}{c} C_{i}^{i^{\prime }}\left(t\right)=I-\left[\varphi_{}^{i}\left(t\right)\times \right]=\left[\begin{array}{ccc} 1 & -\varphi_{z}^{i}\left(t\right) & \varphi_{y}^{i}\left(t\right)\\ \varphi_{z}^{i}\left(t\right) & 1 & -\varphi_{x}^{i}\left(t\right)\\ -\varphi_{y}^{i}\left(t\right) & \varphi_{x}^{i}\left(t\right) & 1 \end{array}\right]. \end{array}$$

Thus, the main problem of the fine alignment turns out to be the estimation of misalignment angles. In order to estimate the misalignment angles, next, the state equation and the measurement equation are established.

The accelerometer's measured specific force projected in the frame *i*′ can be described as follows:

(7) Cbi′f^b=Cbi′[(I+δKA)(I+δA)(−gb+aLAb+aDb)+∇]≈−gi+φi×gi+Cbi∇+Cbi′aLAb+Cbi′aDb +Cbi′(δKA+δA)(−gb+aLAb+aDb).

In consideration of ∇^*b*^ and *a* ^*b*^,

(8) $$\begin{array}{c} \nabla =\nabla^{b}+a^{b}. \end{array}$$

∇^*b*^ and *a* ^*b*^ stand for accelerometer's constant bias and observation white noise, respectively.

Equation (7) can be simplified as follows:

(9) $$\begin{array}{c} C_{b}^{i^{\prime }}{\hat{f}}^{b}+g^{i}-C_{b}^{i^{\prime }}a_{LA}^{b}=\varphi^{i}\times g^{i}+C_{b}^{i}\nabla^{b}+C_{b}^{i}a^{b}+\delta a^{i}. \end{array}$$

Comparing (9) with (7),

(10) $$\begin{array}{c} \delta a^{i}=C_{b}^{i^{\prime }}\left(\delta K_{A}+\delta A\right)\left(-g^{b}+a_{LA}^{b}+a_{D}^{b}\right). \end{array}$$

Integrating (10) from *t*_1_ to *t* yields

(11) ∫t1tCbi′f^b+∫t1tgi−∫t1tCbi′aLAbdτ =∫t1tφi×gidτ+∫t1tCbi∇bdτ+∫t1tCbiabdτ+∫t1tδaidτ.

The measurement vector is

(12) $$\begin{array}{c} Z={\tilde{V}}^{i}=V_{f}^{i}-V_{g}^{i}-V_{LA}^{i}=\delta V^{i}+V_{W}+\delta V_{D}^{i}, \end{array}$$

where

(13) $$\begin{array}{c} V_{f}^{i}=\int_{t_{0}}^{t}C_{b}^{i^{\prime }}{\hat{f}}^{b}d\tau ,\;\;V_{g}^{i}=\int_{t_{0}}^{t}g^{i}d\tau ,\\ V_{LA}^{i}=\int_{t_{1}}^{t}C_{b}^{i^{\prime }}a_{LA}^{b}d\tau =C_{b}^{i^{\prime }}\left(\omega_{ib}^{b}\times r^{b}\right),\\ \delta V_{D}^{i}=\int_{t_{1}}^{t}\delta a^{i}d\tau \\ \delta V^{i}=\int_{t_{1}}^{t}\varphi^{i}\times g^{i}d\tau +\int_{t_{1}}^{t}C_{b}^{i}\nabla^{b}d\tau +\int_{t_{1}}^{t}C_{b}^{i}a^{b}d\tau . \end{array}$$

From (13), we can get the velocity-error equation in the inertial frame

(14) $$\begin{array}{c} \delta {\dot{V}}^{i}\left(t\right)=-g^{i}\left(t\right)\times \varphi^{i}\left(t\right)+C_{b}^{i}\left(t\right)\nabla^{b}+C_{b}^{i}\left(t\right)a^{b}. \end{array}$$

And the misalignment-angle equation can be obtained from the differential equations of *C*_*b*_^*i*^(*t*) and *C*_*b*_^*i*′^(*t*):

(15) $$\begin{array}{c} {\dot{\varphi }}^{i}\left(t\right)=-C_{b}^{i}\left(t\right)\varepsilon^{b}-C_{b}^{i}\left(t\right)\omega^{b}, \end{array}$$

where *ε* ^*b*^ and *ω* ^*b*^ stand for gyro's constant drift and observation white noise, respectively.

Then the state equation can be represented as

(16) $$\begin{array}{c} \dot{X}\left(t\right)=A\left(t\right)X\left(t\right)+B\left(t\right)W, \end{array}$$

where the state vector and the white noises are

(17) X(t)=[δVxi(t),δVyi(t),δVzi(t),φxi(t),φyi(t),φzi(t),  εxb,εyb,εzb,∇xb,∇yb,∇zb]T,W=[axb,ayb,azb,wxb,wyb,wzb,0,0,0,0,0,0]T.

The state matrix and the system noise matrix in (16) are

(18) $$\begin{array}{c} A\left(t\right)=\left[\begin{array}{cccc} 0_{3\times 3} & -\left[g^{i}\left(t\right)\times \right] & 0_{3\times 3} & C_{b}^{i}\left(t\right)\\ 0_{3\times 3} & 0_{3\times 3} & -C_{b}^{i}\left(t\right) & 0_{3\times 3}\\ 0_{3\times 3} & 0_{3\times 3} & 0_{3\times 3} & 0_{3\times 3}\\ 0_{3\times 3} & 0_{3\times 3} & 0_{3\times 3} & 0_{3\times 3} \end{array}\right],\\ B\left(t\right)=\left[\begin{array}{cccc} C_{b}^{i}\left(t\right) & 0_{3\times 3} & 0_{3\times 3} & 0_{3\times 3}\\ 0_{3\times 3} & -C_{b}^{i}\left(t\right) & 0_{3\times 3} & 0_{3\times 3}\\ 0_{3\times 3} & 0_{3\times 3} & 0_{3\times 3} & 0_{3\times 3}\\ 0_{3\times 3} & 0_{3\times 3} & 0_{3\times 3} & 0_{3\times 3} \end{array}\right]. \end{array}$$

From (12), the measurement equation can be described as

(19) $$\begin{array}{c} Z\left(t\right)=HX\left(t\right)+V_{W}+\delta V_{D}^{i}, \end{array}$$

where *δV*_*D*_^*i*^ is uncertainty observation noise and *V*_*W*_ is observation white noise.

The measurement matrix is

(20) $$\begin{array}{c} H=\left[\begin{array}{cc} I_{3\times 3} & 0_{3\times 9} \end{array}\right]. \end{array}$$

## 3. Observability Analyses

For the fine alignment model, the observability is an important issue and requires careful investigation, as it reveals the inherent estimability of the system. In this section, the observability analysis of the fine alignment model was discussed using PWCS theory and SVD theory before designing the filter.

### 3.1. Observability Analysis Using PWCS

What (16) and (19) describe is a time-varying system. The observability of the system can be analyzed by observability analysis of PWCS theory.

According to the PWCS theory, a time varying system can be replaced by piece-wise time invariant system. The period is divided to *r* segments, and each coefficient matrix is approximated to constant in each segment. Then the observability matrix of the PWCS model is

(21) $$\begin{array}{c} Q\left(r\right)=\left[\begin{array}{c} Q_{1}\\ Q_{2}e^{A_{1}\Delta_{1}}\\ Q_{3}e^{A_{2}\Delta_{2}}e^{A_{1}\Delta_{1}}\\ \vdots \\ Q_{r}e^{A_{r-1}\Delta_{r-1}}\cdots e^{A_{1}\Delta_{1}} \end{array}\right], \end{array}$$

where *Q*_*i*_ = [*H* ^*T*^,(*HA*_*i*_)^*T*^,…,(*HA*_*i*_^*n*−1^)^*T*^]^*T*^, Δ_*i*_  (*i* = 1,2,…, *r*) is the time interval between *t*_*i*_ and *t*_*i*+1_.

According to the following theorem, the observability analysis can be performed by a simplified matrix instead of *Q*(*r*).

**Theorem 1**

If Null (*Q*_*i*_) ⊂ Null (*A*_*i*_),  1 ≤ *i* ≤ *r*, then

(22) $$\begin{array}{c} \text{ Null }\left\{Q\left(r\right)\right\}=\text{ Null }\left\{Q_{s}\left(r\right)\right\}\\ \text{ Rank }\left\{Q\left(r\right)\right\}=\text{ Rank }\left\{Q_{s}\left(r\right)\right\}, \end{array}$$

where

(23) $$\begin{array}{c} Q_{s}\left(r\right)=\left[\begin{array}{c} Q_{1}\\ Q_{2}\\ \vdots \\ Q_{r} \end{array}\right]. \end{array}$$

Equation (21) is called total observability matrix (TOM), and (23) is called stripped observability matrix (SOM).  Theorem 1 shows that the observability of a continuous PWCS may be identified through SOM instead of TOM to greatly simplify the computation. If a TOM or SOM is a full-rank matrix, all the states are completely observable, otherwise linear transform can be used to find out the states which are not observable.

### 3.2. Observability Analysis Using SVD

The PWCS theory can only determine whether the system is completely observable or not, but it cannot determine the degree of observability [17]. The observable degree for every state can be computed by means of singular value decomposition method.

According to the SVD theorem, the observability matrix *Q* can be rewritten as follows:

(24) $$\begin{array}{c} Q=U\Sigma V^{T}, \end{array}$$

where *U* and *V* are unitary orthogonal matrices and Σ is a diagonal matrix:

(25) $$\begin{array}{c} U=\left[u_{1},u_{2},\ldots ,u_{m}\right],\;\;V=\left[v_{1},v_{2},\ldots ,v_{m}\right],\\ \Sigma ={\left[\begin{array}{cc} S & O_{(m-r)\times r} \end{array}\right]}^{T}, \end{array}$$

where $S=\mathrm{diag}\{\begin{array}{cccc} \sigma_{1} & \sigma_{2} & \cdots & \sigma_{r} \end{array}\}$, *σ*_1_ ≥ *σ*_2_ ≥ ⋯≥*σ*_*r*_ ≥ 0 and *σ*_*i*_ is the singular value of the *Q* as shown in Table 1.

All the 12 state parameters' observable degrees are large enough to be considered observable. But observable degrees of different states are not the same; it is impossible to confirm which states are more observable and which are less. To establish one-to-one correspondence between the states to the singular values, we will make an analysis below.

The measurement vector *Y* can be written as

(26) $$\begin{array}{c} Y=QX_{0}=\sum_{i=1}^{r}\sigma_{i}\left(v_{i}^{T}X_{0}\right)u_{i}. \end{array}$$

The initial state *X*_0_ can be estimated by

(27) $$\begin{array}{c} X_{0}={\left(U\Sigma V^{T}\right)}^{-1}Y=\sum_{i=1}^{r}\left(\frac{u_{i}^{T}y}{\sigma_{i}}\right)v_{i}. \end{array}$$

According to (27), we can calculate the state corresponding to each singular value.

The degree of the observability of the *k*th state *η*_*k*_ is defined as

(28) $$\begin{array}{c} \eta_{k}=\frac{\sigma_{i}}{\sigma_{0}},\;i=1,2,\ldots ,n,\\ \sigma_{i}\sim \max\left[\frac{u_{i}^{T}yv_{i}}{\sigma_{i}}\right], \end{array}$$

where *σ*_0_ is the singular value of external observable state and *σ*_*i*_ is the singular value which let [*u*_*i*_^*T*^ *yv*_*i*_/*σ*_*i*_]_*k*_ get a maximum.

## 4. Simulation Results 

### 4.1. Foundation of *H*_*∞*_ Filter Equation

For the SINS error equation described as (19), the interference of acceleration is introduced. *δV*_*D*_^*i*^ is uncertainty observation noises, consisting of disturbing velocity caused by the aircraft's pitch and roll. When it comes to random disturbances, the Kalman filter becomes not so effective. Studies show that the advantage of the *H*_*∞*_ filter in comparison with the Kalman filter is that no statistical assumptions on the noises are required, and the filter is more robust when the noises are uncertainties in a system. So the *H*_*∞*_ robust filter is designed in the fine alignment procedure. The error model which we discussed above can be considered as the following state-space system:

(29) $$\begin{array}{c} X_{K}=\phi_{K-1}X_{K-1}+\Gamma_{K-1}W_{K-1},\\ Y_{K}=H_{K}X_{K}+V_{K},\\ Z_{K}=L_{K}X_{K}. \end{array}$$

*L*_*K*_ makes the ratio of estimation error signal energy and disturbance energy less than the prespecified number *γ* [11]. And filtering error is defined as:

(30) $$\begin{array}{c} e_{k}={\hat{Z}}_{K}-L_{K}X_{K}. \end{array}$$

For a fixed positive number *γ* > 0, the job is to find *H*_*∞*_ suboptimal estimation ${\hat{Z}}_{K}=F_{f}(y_{0},y_{1},\ldots ,y_{k})$ to make ||*T*_*k*_(*F*_*f*_)||_*∞*_ < *γ*. It can be shown as

(31) $$\begin{array}{c} \underset{F_{f}}{\inf}\underset{X_{0},W\in h_{2},V\in h_{2}}{\sup}\frac{{||e_{k}||}_{2}^{2}}{{||X_{0}-{\hat{X}}_{0}||}_{P_{0}^{-1}}^{2}+{||W_{k}||}_{2}^{2}+{||V_{k}||}_{2}^{2}}<\gamma^{2}. \end{array}$$

The mathematical equations of *H*_*∞*_ filtering are as follows:

(32) S^K∖K=LKX^K∖K,X^K+1∖K+1=ϕK+1∖KX^K∖K+KK+1(ZK+1−HK+1ϕK+1∖KX^K∖K),KK+1=PK+1HK+1T(HK+1PK+1HK+1T+RK+1)−1,PK+1=ϕK+1∖KPKϕK+1∖KT+ΓKΓKT −ϕK+1∖KPK[HKTLKT]Re,k−1[HKLK]PKϕK+1∖KT,Re,k=[RK00−λ2I]+[HKLK]PK[HKTLKT].

**Figure 1:**
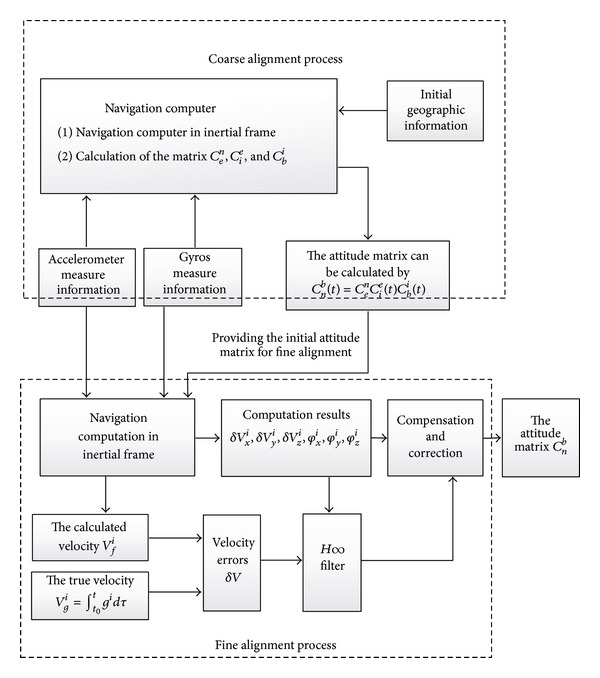
System block diagram.

The framework of the proposed alignment method is shown in Figure 1.

### 4.2. Simulate Conditions

The IMU errors are set as follows: the gyro constant drift: 0.01°/h, the gyro random noise: 0.001°/h, the accelerator bias: 1 × 10^−4^ g, and the accelerator measurement noise: 1 × 10^−5^ g. In the simulation, the plane is assumed to be rocked by the wind gusts, fuel injection, and uncertain human impact. The roll *λ*, pitch *θ*, and heading *ψ*, resulting from the plane's rocking are described as follows:

(33) $$\begin{array}{c} \psi =30^{{}^{\circ}}+5^{{}^{\circ}}\cos\left(\frac{2\pi }{7}t+\frac{\pi }{3}\right),\\ \theta =7^{{}^{\circ}}\cos\left(\frac{2\pi }{5}t+\frac{\pi }{4}\right),\\ \gamma =10^{{}^{\circ}}\cos\left(\frac{2\pi }{6}t+\frac{\pi }{7}\right). \end{array}$$

The velocity caused by heave, surge, and sway is as follows:

(34) $$\begin{array}{c} V_{D_{i}}=A_{D_{i}}\omega_{D_{i}}\cos\left(\omega_{D_{i}}t+\varphi_{D_{i}}\right), \end{array}$$

where *i* = *x*, *y*, *z*, *A*_*D*_*x*__ = 0.02 m, *A*_*D*__*y*_ = 0.03 m, *A*_*D*_*z*__ = 0.3 m, *ω*_*D*_*i*__ = 2*π*/*T*_*D*_*i*__, *T*_*D*_*x*__ = 7 s, *T*_*D*_*y*__ = 6 s, and *T*_*D*_*z*__ = 8 s. *φ*_*D*_*i*__ obeys the uniform distribution on the interval [0, 2*π*].

### 4.3. The Result of Observability Analysis

**Figure 2:**
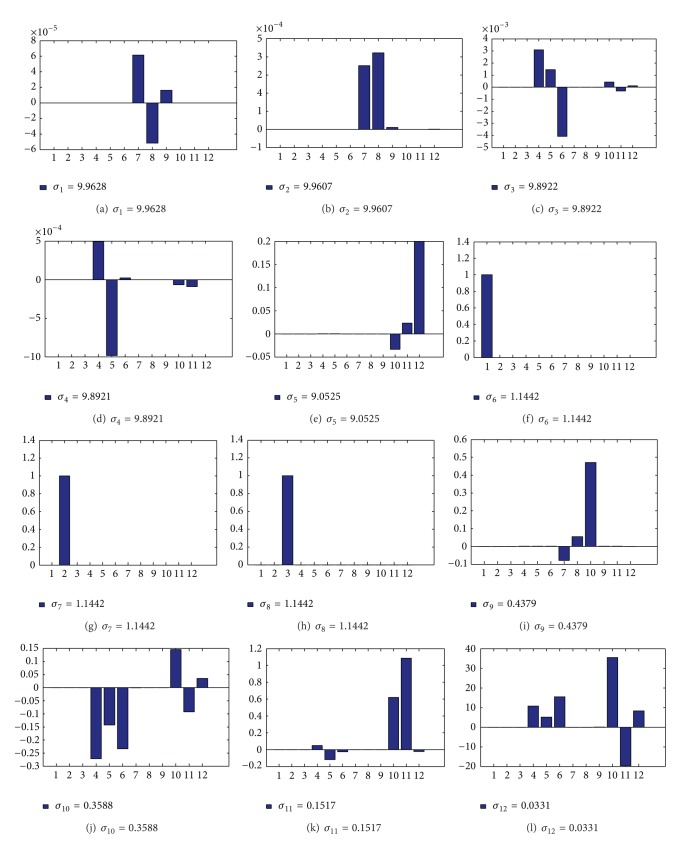
Initial state *X*_0_.

The initial state *X*_0_ corresponding to each singular value can be calculated. Histogram of the state is shown in Figure 2.

Figure 2 shows that the first two singular values are 9.9628 and 9.9607, and the corresponded states are *ε*_*x*_^*b*^ and *ε*_*y*_^*b*^. The third singular value is 9.8922, the corresponded state is *φ*_*z*_^*i*^(*t*), and the fourth singular value which is 9.8921 corresponds to the state *φ*_*y*_^*i*^(*t*). The singular value 9.0522 corresponds to the state ∇_*z*_^*b*^. The observability degree of *δV*_*x*_^*i*^(*t*), *δV*_*y*_^*i*^(*t*) and *δV*_*z*_^*i*^(*t*), is almost 1. Compared with the former eight states, the observability degree of *ε*_*z*_^*b*^(0.4379),  *φ*_*x*_^*i*^(*t*)(0.3588), ∇_*y*_^*b*^(0.1517), and ∇_*x*_^*b*^(0.0331) are a little smaller, but they are large enough to be observable.

Thus the observable degree of the system is computed by SVD theory. It can be seen that the system is completely observable all the 12 system state parameters can be estimated. On the basis of observability, filter has been designed to estimate the system state parameters. In order to prove the analysis results, simulations are carried out as follows.

### 4.4. Simulation of SINS Alignment

**Figure 3:**
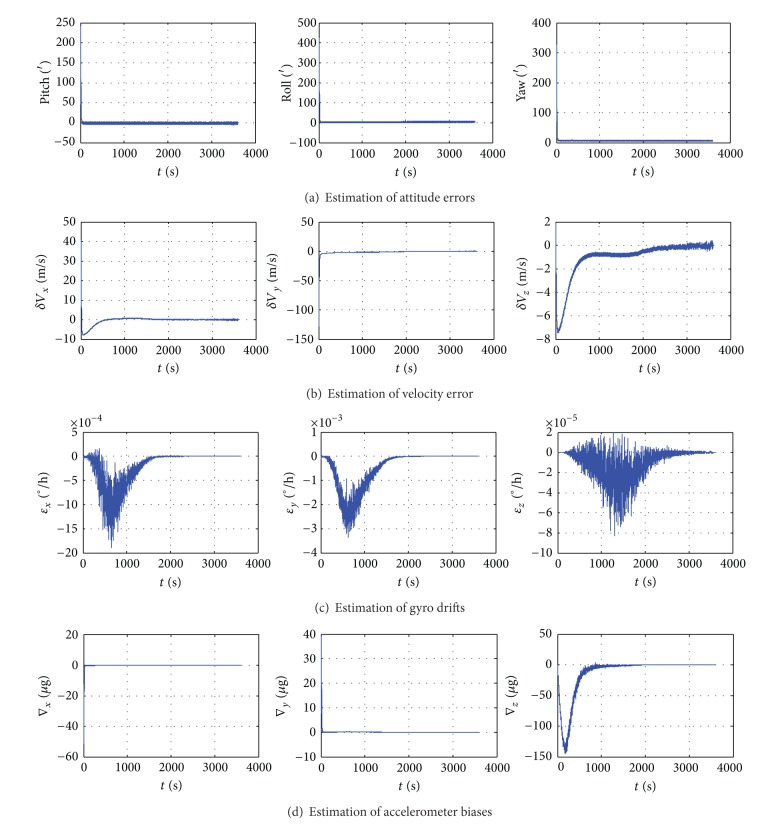
The simulation results of *H*_*∞*_ filtering.

The simulation results are shown in Figure 3. It is clear that all the states are observable; the result confirms the aforementioned analysis.

According to Figure 3(a), convergence time of level misalignment angle is within 60 s, precision for 1′, while azimuth angle convergence time is within 100 s, precision about 4′. The three velocity errors as observed variables depicted in Figure 3(b) are certainly observable. As seen from Figure 3(c), gyro drifts converge not so rapidly, but a rather good filtering result can be obtained.  Figure 3(d) shows that the horizontal accelerometers constant biases ∇_*x*_ and ∇_*y*_ converge to a constant value within a short time. The vertical converges soon afterwards with good estimation performance. The simulation proves the analysis results that all the system state parameters can be estimated by *H*_*∞*_ filter.

It can be seen that the alignment method can effectively deal with the random disturbances in the dynamic environment. Using *H*_*∞*_ filter can improve the system sensitivity to noise and the divergence speed of azimuth.

## 5. Conclusion

The aircraft SINS is prone to violent rocking, which makes the initial alignment difficult. In this paper the gravity in the inertial frame as a reference is used, so as to counteract the disturbed components. With the deduced SINS model on disturbances base, the relevant *H*_*∞*_ filter is designed. Before the filter is designed, observability is analyzed by PWCS theory and SVD theory; degree of observability for every state can be computed. The simulation shows that the alignment method can effectively deal with the random disturbances in the dynamic environment. And the system is completely observable; all the system state parameters can be estimated by *H*_*∞*_ filter.

## Figures and Tables

**Table 1:** Singular values of system observable matrix *Q*.

*σ*_1_ = 9.9628	*σ*_2_ = 9.9607	*σ*_3_ = 9.8922
*σ*_4_ = 9.8921	*σ*_5_ = 9.0525	*σ*_6_ = 1.1442
*σ*_7_ = 1.1442	*σ*_8_ = 1.1442	*σ*_9_ = 0.4379
*σ*_10_ = 0.3588	*σ*_11_ = 0.1517	*σ*_12_ = 0.0331
